# Isoniazid-induced seronegative cutaneous lupus in a patient with lupus vulgaris

**DOI:** 10.1016/j.jdcr.2026.07.010

**Published:** 2026-07-10

**Authors:** Sandesh Shah, Joshana Shrestha, Mohan Bhusal, Deepika Neupane, Radhika Maharjan, Rabin Baniya

**Affiliations:** a Consultant Dermatologist, Department of Dermatology, Nepal Medical College and Teaching Hospital, Kathmandu, Nepal; b Resident Doctor, Department of Dermatology, Nepal Medical College and Teaching Hospital, Kathmandu, Nepal; c Lecturer, Department of Prosthodontics, B.P Koirala Institute of Health Sciences, Dharan, Nepal

**Keywords:** antitubercular therapy, cutaneous lupus erythematosus, drug-induced lupus, isoniazid, lupus vulgaris, seronegative

## Introduction

Drug-induced lupus erythematosus (DILE) is an autoimmune syndrome triggered by prolonged exposure to certain medications and accounts for approximately 6% to 12% of all lupus cases.^1^ Although the classic presentation resembles systemic lupus erythematosus, with positive antinuclear antibody (ANA) and antihistone antibody tests, drug-induced cutaneous lupus erythematosus (DI-CLE) is a less common and more heterogeneous variant.^2^

Unlike systemic DILE, cutaneous variants may show variable or even absent serologic markers, particularly in subacute cutaneous lupus erythematosus (SCLE)-like presentations.^2^^,^^3^ Isoniazid, a first-line antitubercular agent, is a recognized cause of drug-induced lupus, although most reported cases describe systemic manifestations with positive serology.^1^^,^^4^ Seronegative cutaneous presentations are uncommon and may pose significant diagnostic challenges.

We report a case of seronegative drug-induced cutaneous lupus erythematosus that developed during treatment of lupus vulgaris, highlighting the paradoxical coexistence of 2 distinct “lupus” entities and emphasizing the importance of clinicopathological correlation and therapeutic dechallenge in establishing causality. Recognition of immune-mediated adverse reactions during antitubercular therapy is essential to ensure both patient safety and uninterrupted tuberculosis management.

## Case report

A 41-year-old female presented with an asymptomatic lesion on the bridge of the nose of 1 year's duration. The lesion had an insidious onset and was gradually progressive, later developing central atrophy. There were no associated pain, discharge, photosensitivity, oral ulcers, arthralgia, or constitutional symptoms. There was no personal or family history of tuberculosis.

Cutaneous examination revealed a well-defined erythematous plaque measuring approximately 5 × 2 cm over the bridge of the nose with central atrophy and ill-defined margins (Fig 1, *A*). No sensory loss, nerve thickening, or regional lymphadenopathy was noted. No other cutaneous or mucosal lesions were present at initial presentation. Differential diagnoses considered clinically included lupus vulgaris, discoid lupus erythematosus, cutaneous leishmaniasis, sarcoidosis, and granulomatous rosacea.

**Fig 1:**
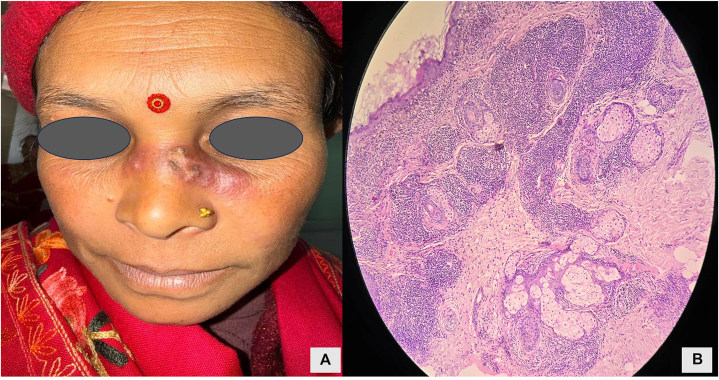
**A,** Erythematous plaque with central atrophy over the bridge of the nose at presentation. **B,** Skin biopsy showing dermal granulomatous inflammation composed of epithelioid cells and lymphocytes (hematoxylin and eosin stain, 10×).

Investigations showed a positive tuberculin skin test, with normal chest radiography. A skin biopsy from the nasal plaque showed orthokeratosis and focal thinning of the epidermis with flattening of rete ridges. The dermoepidermal junction demonstrated pigmentary incontinence. The dermis revealed aggregates of epithelioid cells admixed with lymphocytes forming ill-defined granulomas, along with a dense lymphohistiocytic infiltrate predominantly composed of lymphocytes extending into the subcutis (Fig 1, *B*). No Leishman-Donovan bodies were identified. Based on clinicopathological correlation, a diagnosis of lupus vulgaris was established.

The patient was initiated on first-line antitubercular therapy (ATT) as per national tuberculosis treatment guidelines.

Approximately 1 month after initiation of ATT, the patient developed progressive symmetric erythema over the cheeks, sparing the nasolabial folds and extending to the ears (Fig 2). The eruption was associated with marked photosensitivity, such that the patient was unable to tolerate even 5 minutes of sun exposure. There was no involvement of the neck, trunk, or extremities. There were no visual symptoms suggestive of ethambutol toxicity, features of hepatotoxicity, peripheral neuropathy, or other systemic adverse effects attributable to antitubercular therapy. No mucosal lesions, systemic symptoms, or exacerbation of the nasal plaque were noted; in fact, the lupus vulgaris lesion showed clinical improvement.

**Fig 2:**
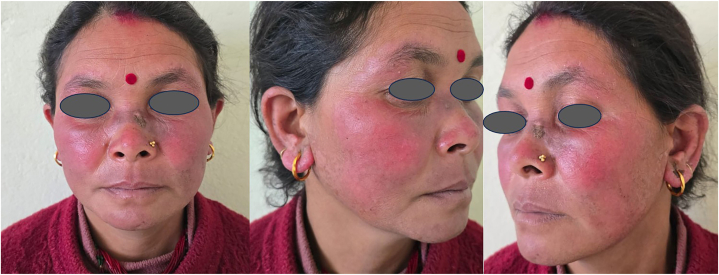
Symmetrical erythema over the malar areas with sparing of the nasolabial folds and involvement of the ears following initiation of antitubercular therapy.

Given the temporal association with ATT and the characteristic photo-distributed erythema, drug-induced cutaneous lupus was suspected. Antinuclear and antihistone antibody testing was performed and was negative. An extended ANA profile, including extractable nuclear antigen antibodies, was not performed because of financial constraints. A repeat skin biopsy of the erythematous malar area revealed follicular plugging, flattening of rete ridges, basal cell vacuolar degeneration, and pigment incontinence. The dermis showed edema with perivascular and periadnexal lymphohistiocytic infiltrate (Fig 3). These histopathological features were consistent with cutaneous lupus erythematosus.

**Fig 3:**
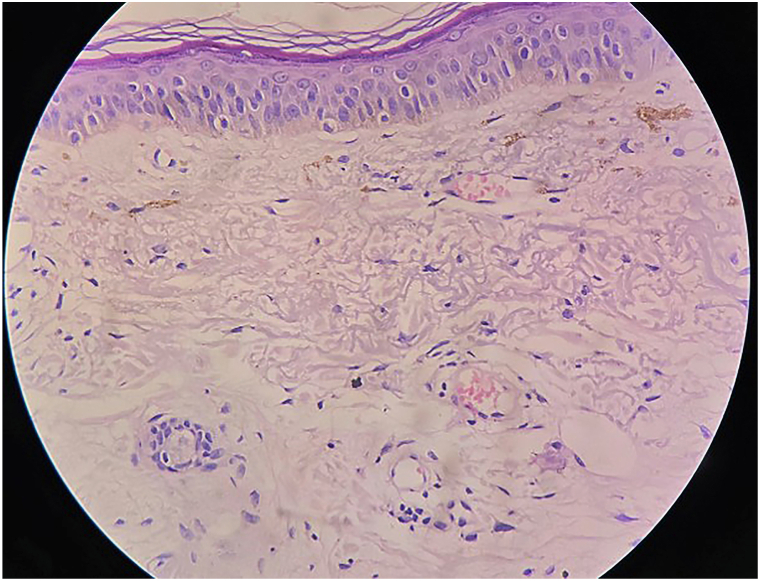
Showing basal cell vacuolar degeneration, pigment incontinence, perivascular and periadnexal lymphohistiocytic infiltrate in the dermis (hematoxylin and eosin stain, 20×).

Given the clinical, temporal, and histopathological findings, isoniazid-induced seronegative cutaneous lupus erythematosus was considered. Isoniazid was discontinued, and the patient was continued on rifampicin, pyrazinamide, and ethambutol, with levofloxacin added as part of the modified antitubercular regimen according to national guidelines. She was treated with topical mometasone furoate cream twice daily for 2 weeks, then once daily for another 2 weeks (Fig 4), along with oral levocetirizine 5 mg nightly for 2 weeks, and strict photoprotection with a broad-spectrum mineral sunscreen.

**Fig 4:**
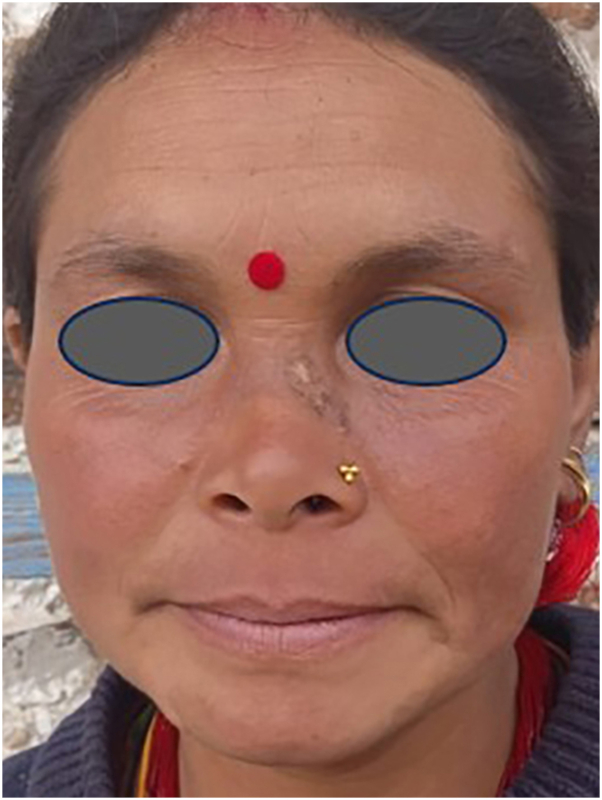
Follow-up at 1 month showing resolution of the facial erythema after stopping isoniazid.

At 1-month follow-up, there was marked improvement in facial erythema, with complete resolution of photosensitivity. No new lesions were noted. The patient continues antitubercular therapy without isoniazid and remains under regular follow-up.

## Discussion

This case presents a striking clinical paradox: a patient treated for lupus vulgaris subsequently developed a lupus-like cutaneous eruption attributable to the therapy itself. The coexistence of infectious lupus (lupus vulgaris) and autoimmune drug-induced cutaneous lupus within the same therapeutic timeline underscores the complexity of dermatologic diagnosis.

Drug-induced lupus erythematosus is typically associated with positive ANA and antihistone antibodies, particularly in systemic cases.^1^ Nonetheless, drug-induced cutaneous lupus differs in both immunological and clinical aspects. Grönhagen et al showed that drug-induced SCLE can present with diverse serologic profiles, with ANA negativity observed in some cases.^2^ Likewise, Lowe et al reported cases of isoniazid-induced SCLE with negative ANA and antihistone antibodies, highlighting that seronegativity does not rule out the diagnosis.

DILE's pathogenesis involves multiple factors. Proposed mechanisms include impaired DNA methylation in CD4+ T cells, leading to autoreactivity; drug metabolite–mediated neoantigen formation; and genetic factors, such as slow acetylator status related to isoniazid metabolism.^1^^,^^5^ Ultraviolet radiation also plays a role by increasing keratinocyte apoptosis and exposing nuclear antigens, which may explain the intense photosensitivity observed in cutaneous variants.^3^ Seronegative presentations may reflect a predominantly skin-focused immune response that does not produce persistent systemic autoantibodies.^2^^,^^6^

Antitubercular drugs are associated with a spectrum of cutaneous adverse reactions, including lichenoid eruptions, maculopapular eruptions, fixed drug eruptions, and, rarely, severe reactions such as Stevens–Johnson syndrome and toxic epidermal necrolysis. Consequently, any new skin eruptions during antitubercular treatment should be carefully investigated to determine the cause and inform proper management.^7^ Several differential diagnoses were considered. Dissemination of lupus vulgaris was unlikely given the absence of granulomatous inflammation in the facial erythematous lesions and the concurrent regression of the primary nasal plaque. Discoid lupus erythematosus was considered; however, the abrupt onset following initiation of antitubercular therapy and rapid improvement after withdrawal favored a drug-induced etiology. Cutaneous leishmaniasis was excluded histologically due to the absence of Leishman–Donovan bodies and the lack of parasitized macrophages. Phototoxic or photoallergic dermatitis was less likely given the presence of basal vacuolar degeneration, follicular plugging, and periadnexal lymphohistiocytic infiltrate—features characteristic of cutaneous lupus erythematosus.^3^

The temporal link was strong: the eruption appeared 1 month after starting therapy and then worsened. Isoniazid, a common first-line antitubercular drug, is known to be associated with drug-induced lupus.^1^^,^^4^ Stopping isoniazid while continuing other medications resulted in significant improvement within a month and complete resolution of photosensitivity. A limitation of this case is that topical mometasone furoate was initiated concurrently with isoniazid withdrawal, making it difficult to completely distinguish the contribution of drug discontinuation from that of topical anti-inflammatory therapy to the observed clinical improvement. Additionally, an extended extractable nuclear antigen profile was not performed because of financial constraints, which may have provided further immunologic characterization of the eruption. Nevertheless, the close temporal relationship between isoniazid exposure and symptom onset, histopathological findings consistent with cutaneous lupus erythematosus, and improvement following isoniazid withdrawal support a probable diagnosis of isoniazid-induced seronegative cutaneous lupus erythematosus.

According to the Naranjo scale for adverse drug reactions, this reaction is rated as “probable.”^8^ In tuberculosis management, interruption of first-line therapy can have serious consequences, such as treatment failure and drug resistance. Differentiating drug-induced autoimmune phenomena from progression of infection or unrelated dermatoses is therefore critical. In our case, identifying the adverse reaction enabled us to carefully stop isoniazid while continuing the other medications, avoiding an unnecessary interruption of antitubercular therapy. This highlights the need to effectively manage side effects without compromising the overall treatment of tuberculosis.

## Conclusion

This case highlights the need to recognize drug-induced cutaneous lupus as a possible side effect of isoniazid therapy, even when serologic markers are absent. In areas where tuberculosis is common and antitubercular therapy is frequently used, new photo-distributed eruptions during treatment should lead clinicians to consider drug-induced autoimmunity. Careful clinical and pathological evaluation, prompt recognition and withdrawal of the suspected drug, together with appropriate supportive treatment, may result in clinical improvement. Understanding seronegative cases is crucial to prevent misdiagnosis and unnecessary treatment disruptions in effective tuberculosis management.

## Conflicts of interest

None disclosed.
